# Resting Level of Insulin-Like Growth Factor-1 Is Not at Play in Cardiac Enlargement in Endurance-Trained Adolescents

**DOI:** 10.1155/2019/9647964

**Published:** 2019-09-30

**Authors:** Louise Rundqvist, Jan Engvall, Peter Blomstrand, Emma Carlsson, Maria Faresjö

**Affiliations:** ^1^Department of Natural Science and Biomedicine, School of Health and Welfare, Jönköping University, Jönköping, Sweden; ^2^Department of Clinical Physiology, Linköping University, Linköping, Sweden; ^3^Centre for Medical Image Science and Visualization (CMIV), Linköping University, Linköping, Sweden; ^4^Department of Clinical Physiology, Region Jönköping County, Jönköping, Sweden

## Abstract

**Purpose:**

The study aimed to investigate resting levels of several selected growth and metabolic hormones in a group of 24 endurance-trained adolescents (aged 13–19 years) compared with 24 untrained age- and sex-matched controls, and to investigate if increased cardiac dimensions were related to these hormones at rest with emphasis on insulin-like growth factor-1 (IGF-1).

**Methods:**

The hormones (cortisol, IGF-1, IGF-2, follicle-stimulating hormone, growth hormone, luteinizing hormone, prolactin, and thyroid-stimulating hormone) were analysed with chemiluminescence microparticle immunoassay (CMIA) or multiplex fluorochrome (Luminex) technique. Cardiac dimensions were assessed by echocardiographic examination at rest. Peak oxygen uptake was obtained by a maximal cardiopulmonary exercise test on a treadmill.

**Results:**

Circulating levels of analysed hormones at rest did not differ between the groups. A correlation was found between increased cardiac dimensions and IGF-1 in the controls, but not in the active group. This correlation declined also among the controls when the cardiac parameters were indexed for body surface area.

**Conclusion:**

Increased cardiac dimensions in endurance-trained adolescents could not be related to resting levels of hormones associated with growth and metabolism, including IGF-1 and GH. In addition, the resting levels of these hormones seem not to be affected by intense regular endurance exercise in adolescents. These findings may contribute to the knowledge about cellular signaling that trigger growth as well as cardiac adaptation to endurance training in young athletes.

## 1. Introduction

Cardiac adaptation to long-term endurance exercise is characterized by increased chamber size and wall thickness, which in turn is associated with increased aerobic capacity, in both adults and adolescents [1–3]. Signals that trigger cardiac hypertrophy include increased afterload and gene expression of several growth factors. Cellular signaling pathways differ between physiological cardiac hypertrophy (athlete's heart) and pathological hypertrophy (cardiac compensation in the setting of disease such as hypertension and valvular heart disease). Pathological hypertrophy has been associated with reactivation of the fetal cardiac gene programme, which means activation of genes that are expressed in the fetal ventricles during development but repressed after birth [4, 5]. This phenomenon, however, does not occur in physiological hypertrophy. The best characterized intracellular signaling cascades responsible for physiological hypertrophy are supposed to be activated by the circulating hormone insulin-like growth factor 1 (IGF-1) [6, 7]. Along with thyroid hormones (including thyroid-stimulating hormone [TSH]), cortisol, and gonadal steroid hormones (e.g., estradiol and testosterone), IGF-1 is crucial for the hormonal control of growth during childhood and adolescence [8]. Particularly in the heart, in addition to the cellular signaling pathway responsible for inducing physiological cardiac hypertrophy, IGF-1 also promotes formation of cardiomyocytes and protects against cell death [7, 9, 10]. IGF-1 is secreted mainly by the liver in response to growth hormone (GH) stimulation, which is called the GH–IGF-1 axis [9]. High-intensity exercise has long been known to be a potent stimulus of the GH–IGF-1 axis in both prepubertal and adolescent subjects [11]. We therefore hypothesized that higher resting values of circulating IGF-1 and/or GH may be detected in regular endurance-trained adolescents with increased cardiac dimensions compared with a nontrained control group with comparatively lower cardiac dimensions.

Moreover, in normal puberty, release of hormones with the primary function to regulate bodily growth changes dramatically with maturation in both sexes [12]. The anterior pituitary gland is a major endocrine gland which responds to exercise by increasing the release of GH, prolactin, and TSH and increases the release of follicle-stimulating hormone (FSH) and luteinizing hormone (LH) to a lesser extent [13]. Exercise-associated effects on anabolic hormones promoting puberty-related growth and development may influence the time course of puberty, especially if exercise is maintained for a long time [11]. However, potential effects of training on resting levels of circulating hormones related with growth and metabolism in endurance-trained adolescents are not known. Therefore, in the present study we investigated resting levels of eight circulating hormones related to growth and metabolism in regular endurance-trained adolescents and in an untrained control group individually matched by age and sex. In the same subjects, we also examined whether increased cardiac dimensions were positively related to higher serum levels of these hormones, with emphasis on the growth factor IGF-1.

## 2. Materials and Methods

### 2.1. Study Design and Subjects

This study, a part of the Exercise Project at Jönköping University, Sweden, used a cross-sectional and comparative design. Subjects in the active group consisted of 24 adolescents 13–19 years old, which were recruited from orienteering and cross-country ski clubs in an area of southern Sweden. They were practicing endurance exercise (orienteering, running, cross-country skiing, and cycling) and competed at an elite level in their sports for at least two years prior to study enrollment; on average, they exercised 5 days a week for 30–120 minutes on each occasion in moderate to vigorous intensity, in addition to participation in compulsory physical education in school. The control group was recruited from public schools in the same area, consisted of 24 individually age- and sex-matched adolescents not engaged in regular exercise, except for mandatory physical education at school. Each group included 14 boys and 10 girls, respectively. All subjects were instructed to refrain from exercise on the day the test was performed. They also completed a questionnaire regarding exercise habits and medical conditions. Body surface area (BSA) was calculated according to the DuBois formula.

Written informed consent was obtained from all subjects, and the study was approved by the Regional Ethical Review Board in Linköping, Sweden (Dnr 2013/89–31). The study was executed in accordance with the Declaration of Helsinki.

### 2.2. Procedures

A resting 12-lead electrocardiogram (ECG) was obtained with the MAC 5500HD version 10 (GE Healthcare, Milwaukee, WI, USA). Right arm systolic and diastolic blood pressure was acquired with the subjects in a supine position after at least 5 minutes of rest.

#### 2.2.1. Hormone Analysis

Nonfasting, resting venous blood samples were drawn between 3.45 and 4.15 pm into sample tubes without anticoagulants. The samples were separated, and serum was stored at −80°C until analysis. Nonfasting cortisol in serum was determined with chemiluminescence microparticle immunoassay (CMIA) using Architect i2000 (Abbott, Chicago, Illinois, USA). Cortisol in the sample was bound to anticortisol coated microparticles. After incubation, cortisol acridinium-labeled conjugate was added to the sample of mixture, which was bounded to available binding sites on the anticortisol coated microparticles. The resulting chemiluminescent was measured as relative light units. The detection limit for the assay was 25 nmol/L.

IGF-1, IGF-2, FSH, GH, LH, prolactin, and TSH were analyzed in serum with multiplex fluorochrome technique (Luminex, Bio-Rad Laboratories, USA). All hormones were analyzed by using a human metabolic assay (Bio-Rad Laboratories), and a Bio-Plex 200^TM^ system (Luminex xMAP^TM^ Technology) was used for identification and quantification of each hormone. Median fluorescence intensity (MFI), for each sample was registered and analyzed with Bio-Plex Manager^TM^ Software 5.0. The sample concentration values were estimated from a five-parameter logistic equation based standard curve. The cutoff value for minimum detectable concentrations for each metabolic marker was as follows: IGF-1 (0.29 ng/mL), IGF-2 (0.05 ng/mL), FSH (0.04 mLU/mL), GH (0.03 ng/mL), LH (0.13 mLU/mL), prolactin (0.04 ng/mL), and TSH (0.02 *μ*LU/mL).

#### 2.2.2. Echocardiography and Image Analysis

All participants underwent echocardiographic examination at rest, before the CPET, which previously has been described in detail by Rundqvist et al. [2]. Briefly, a state-of-the-art ultrasound scanner (Vivid E9, GE Healthcare, Horten, Norway) equipped with an M5S probe was used. Cardiac dimensions were obtained by two-dimensional (2D) echocardiographic images acquired from the parasternal long- and short-axis views as well as the apical long-axis and two- and four-chamber views. Additionally, a modified right ventricular- (RV-) focused apical four-chamber view was obtained. LV mass was calculated with the linear method at the parasternal long-axis approach, and the left atrial (LA) volume was calculated by the biplane method of disks [14]. Assessment of the LA parasternal diameter was made by M-mode tracing using the leading edge to leading edge convention. The RV diameter was measured in the inflow tract (RVD1). Right atrial (RA) dimensions were obtained in the 2D apical modified four-chamber view by measuring the area at end systole and the RA diameter between the lateral wall and septum at the midatrial level [14]. All echocardiographic data were analysed offline by a single operator using EchoPAC PC version 110.0 (GE Healthcare, Horten, Norway).

#### 2.2.3. Cardiopulmonary Exercise Test (CPET)

To assess peak VO_2_, CPET on a treadmill was performed by all subjects, where exhaled air was analysed on a breath-by-breath basis for O_2_ and CO_2_ content with a Jaeger Oxycon Pro (VyAir Inc, Mettawa, Ill, USA). A non-rebreathing valve was connected to a mouthpiece to prevent mixing of inspired and expired air. The CPET was according to the modified Bruce protocol [15], which starts with a speed of 2.7 km/h at a grade of 10%, then speed and grade increased every third minute according to the protocol until exhaustion. This protocol is suitable for both children and adults and is recommended when >12 minutes of exercise is expected [16]. We selected this protocol because we wanted to use the same approach for all participants regardless of exercise history, sex, or age. Peak VO_2_ was averaged from the two highest, consecutive measurements immediately before exercise was terminated. Criteria for termination of exercise were exhaustion and/or a respiratory exchange ratio equal to or exceeding 1.1.

### 2.3. Statistics

Statistics were performed with SPSS Statistics software version 21 (IBM software, Armonk, New York, USA). Variables are presented as median value and range (min-max). Differences between the active group and controls were tested with the nonparametric Wilcoxon matched-pairs signed-rank test, with a probability level of <0.05 as significant. Correlation between hormones and cardiac dimensions was analysed by bivariate correlations, and when separated into an active group and controls, linear regression analysis with and without controlling for BSA was performed.

## 3. Results

All resting ECG were normal, none of the subjects reported a history of cardiovascular disease, and all subjects were nonsmokers. The only medical treatment reported was the regular use of bronchodilators to combat asthma in five active and one control participant. Characteristics were similar between the groups except for higher peak VO_2_ and lower resting heart rate (HR) in the active group (Table 1).

There were no differences observed between the groups regarding resting levels of circulating hormones except for prolactin, which was slightly higher in the active group compared with controls (Table 2). When separated according to sex, differences between boys in the active group and controls were detected regarding LH and prolactin; otherwise, no differences could be demonstrated in the subgroups.

Cardiac dimensions of the active group versus controls are presented in Table 3. Increased cardiac size and volumes in all four chambers as well as greater LV mass were found in the active group compared with the controls.

Analysing all participants together, IGF-1 was positively associated to cardiac dimensions such as LVID, LVM, LA diameter, LA volume, RA diameter, and RA area, as presented in Table 4. Also, FSH was correlated to the LA diameter. When comparing the active group with controls, we found associations between IGF-1 and cardiac dimensions only in the group of controls with respect to LVID, LVPWT, LVM, RA diameter, and RA area. No such correlation could be demonstrated in the active group. However, when controlled for BSA the correlation disappears even in the controls (Table 5). No association could be found between the cardiac dimensions and the other hormones (cortisol, IGF-2, GH, LH, prolactin, and TSH).

## 4. Discussion

This is the first study to our knowledge that has analysed the aspects of circulating hormones at rest and their association to cardiac dimensions in endurance-trained adolescents as well as in untrained age- and sex-matched controls. Our findings showed that resting levels of the analysed hormones, including IGF-1 and GH, uncontrolled for any confounding factors, could not be related to greater cardiac dimensions in endurance-trained adolescents. Notably, an association between resting levels of IGF-1 and cardiac dimensions was found in the controls, but it failed to reach significance when controlled for BSA. We found similar levels between the study groups regarding hormones associated to growth and metabolism at rest.

It has previously been confirmed that long-term endurance exercise leads to remodelling of all four cardiac chambers with respect to myocardial mass, wall thickness, and internal diameter in athletic adolescents and adults [1–3]. Biomechanical (i.e., receptors sensitive for changes in volume or pressure load) as well as hormonal mechanisms are dependent factors for cardiac hypertrophy, but knowledge is lacking about how much each factor contributes to cardiac remodelling [4, 17]. The signaling pathway by IGF-1 has been shown to have a key role in physiological cardiac hypertrophy because of its ability to regulate several cellular processes in the heart including growth, metabolism, and apoptosis [6, 9]. Due to the absence of an association between increased cardiac dimensions and resting circulating levels of growth factors such as GH and IGF-1 in the active group in our study, we may speculate that the autocrine impact of locally synthesized IGF-1 and/or the biomechanical mechanism, both at play during regular exercise, are of greater importance for increased atrial and ventricular size than resting levels of growth factors in athletes. The correlation that was found between IGF-1 and cardiac dimensions in the untrained controls may suggest that IGF-1 is of importance for general cardiac growth but fails to trigger cardiac enlargement in athletes. However, when controlling for BSA, no correlation was found even in the controls which may indicate that body size is a factor of great importance for ordinary cardiac growth during adolescence. This is in agreement with a previous study by Janz et al. who reported that predictability in LVM most likely is attributable to normal growth regulated by both genetic and hormonal influences, in healthy children as well as in adolescents [18]. In addition, the authors suggested that body size, particularly fat-free body mass, is an important determinant of heart size and heart growth during adolescence, and that changes in aerobic fitness, presumably attributable to improvements in cardiac function will affect cardiac growth, in agreement with our results.

Although IGF-1 is mainly synthesized and secreted by the liver, other tissues such as the myocardium have the capacity to produce IGF-1, which then acts locally as an autocrine and a paracrine hormone [9]. A prior study in adults showed higher levels of resting cardiac IGF-1 in athletes compared to controls, and they concluded that cardiac IGF-1 in combination with enhanced sympathetic activity was associated with physiological cardiac adaptation in athletes [7]. That study analysed IGF-1 locally from coronary sinus blood and not from systemic veins, and additionally, their subjects were adults and not adolescents, both factors important for the understanding of the results. Samples from coronary sinus blood at rest may have contained cardiac locally secreted IGF-1 which when diluted with circulating blood, would not be possible to detect in the systemic circulation in our participants.

The response of IGF-1 to exercise is fast and peaks approximately 10 minutes after onset of exercise [19], which supports the notion that this substance is difficult to detect under resting conditions. One previous study has, however, demonstrated an increased level of circulating IGF-1 at rest in resistant-trained women, which was not found in age- and body mass-matched endurance-trained athletes [20]. That is consistent with our results on endurance-trained adolescents compared to controls and suggests that resistance but not endurance training may modify the circulating IGF-1 signaling system in a more pronounced way. Moreover, under resting conditions Ubertini et al. did not find any difference in circulating levels of IGF-1 between young elite athletes and sedentary controls [21]. On the other hand, they reported higher levels of GH in athletes who perform very intense training in excess of 12 hours/week compared to controls and athletes training 3–9 hours/week. Thus, resting levels of GH seem to be affected by the intensity of training. In our study, we did not divide the active group into subgroups in respect to the hours of training per week because of the sample size, which may have hidden potential differences in GH levels within the group.

Even if circulating level of IGF-1 has been reported to decrease during periods of heavy training, sports with similar training intensity throughout the season have not found changes in IGF-1 levels over time [11]. In this study, we did not consider whether the data collection was performed during the competitive season or not. However, our questionnaire revealed that subjects in the active group did not change their amount of exercise preseason nor in the competitive season.

The period of adolescence is difficult to define in chronological years because it varies in onset and termination. The present research was performed on adolescents aged 13–19 years, which theoretically means that some of the subjects may have been in a prepubertal phase. Thus, a weakness of our investigations was that we did not take into account maturity status or Tanner stages during the data collection. Obviously, adjustments to these factors would have contributed to a deeper understanding of our results. However, hormones responsible for inducing puberty (e.g., LH and FSH) did not differ significantly at group levels between the endurance-trained and the control group, which may suggest comparable pubertal status between the two groups. There is a large degree of interindividual variation in hormone response to exercise, even when the subjects are matched for age, sex, body composition, and so on [22]. This could theoretically have an effect on circulating resting hormone levels; hence, complicate the interpretation of differences in hormone concentration between individuals. During childhood and puberty there is a spontaneous increase in anabolic hormones promoting puberty-related growth and development. Since exercise maintained for long time has been reported to have effects on these hormone levels [11], exercise may influence puberty and impact the resting levels of circulating hormones in adolescents who practice intense and regular endurance exercise compared with nontrained controls. Although, we could not prove significant differences in hormone levels between the study groups, and to our knowledge, there is a lack of similar previous studies in trained and untrained adolescents. Since our study has a cross-sectional design based on resting levels of hormones, further studies are needed on temporal changes of hormones in relation to exercise.

In conclusion, increased cardiac dimensions in endurance-trained adolescents were not associated with resting circulating levels of growth factors, including IGF-1 and GH, which indicate that other mechanisms and triggers are of greater importance to physiological cardiac hypertrophy in endurance-trained adolescents. In addition, resting levels in serum of hormones associated with growth and metabolism did not differ between the active group and the controls, where the active group had greater cardiac size compared with the controls. Our results may contribute to the knowledge about factors that may (or may not) trigger cardiac hypertrophy in adolescent athletes.

## Figures and Tables

**Table 1:** Characteristics of the study population.

	Active group (*n* = 24)	Controls (*n* = 24)	*P* value
Age (years)	15.5 (13–19)	15.4 (13–19)	0.934
BSA (m^2^)	1.69 (1.37–1.98)	1.72 (1.26–2.23)	0.578
SBP (mmHg)	120 (105–155)	115 (105–130)	0.449
DBP (mmHg)	65 (50–85)	65 (55–80)	0.610
HR at rest (beats/min)	62 (42–85)	69 (49–88)	**0.029**
Peak VO_2_ (mL/kg/min)	62 (53–79)	44 (27–62)	**<0.001**

Data are presented as median with range (min-max). Bold styling denotes statistical significance. BSA, body surface area; SBP and DBP, systolic and diastolic blood pressure; HR, heart rate; VO_2_, oxygen uptake.

**Table 2:** Resting levels of circulating hormones in the active group vs controls and at subgroups of boys (b) and girls (g).

	Active group (*n* = 24, 14 boys and 10 girls)	Control group (*n* = 24, 14 boys and 10 girls)	*P* value
Cortisol (nmol/L)	136	120	0.421
b	125 (40–277)	120 (33–286)	0.541
g	149 (48–305)	108 (37–330)	0.971
IGF-1 (ng/mL)	25	23	0.288
b	27 (11–40)	25 (14–48)	0.427
g	21 (11–43)	19 (4–29)	0.529
IGF-2 (ng/mL)	1.0	0.0	0.134
b	1.1 (0.0–2.8)	0.0 (0.0–8.0)	0.454
g	0.5 (0.0–5.0)	0.0 (0.0–3.0)	0.315
FSH (mLU/mL)	7.4	5.7	0.177
b	6.3 (1.8–12.1)	4.1 (1.4–9.1)	0.137
g	7.8 (3.6–17.9)	6.3 (3.8–11.9)	0.684
GH (ng/mL)	1.4	2.0	0.327
b	1.5 (0.1–26.1)	3.2 (0.2–28.6)	0.571
g	1.1 (0.6–6.4)	1.8 (0.5–13.8)	0.315
LH (mLU/mL)	2.1	1.4	0.095
b	2.2 (0.7–11.5)	0.7 (0.0–4.6)	**0.031**
g	2.1 (1.2–28.0)	2.0 (0.6–6.1)	0.796
Prolactin (ng/mL)	1.5	0.9	**0.049**
b	1.6 (0.3–4.8)	0.9 (0.0–4.3)	**0.039**
g	1.2 (0.3–5.5)	0.9 (0.0–2.8)	0.481
TSH (*μ*LU/mL)	2.6	2.8	0.877
b	2.7 (1.1–10.6)	2.8 (1.2–12.0)	0.910
g	2.6 (0.8–11.0)	2.5 (1.3–9.8)	0.796

Data are presented as median with range (min-max). Bold styling denotes statistical significance. IGF, insulin-like growth factor; FSH, follicle-stimulating hormone; GH, growth hormone; LH, luteinizing hormone; TSH, thyroid-stimulating hormone.

**Table 3:** Cardiac parameters of the active group and controls.

	Active group (*n* = 24)	Controls (*n* = 24)	*P* value
LVID (mm)	49 (44–56)	45 (41–54)	**0.003**
LVPWT (mm)	7.6 (6.1–11.8)	7.0 (5.3–9.3)	**0.006**
LVM (g)	109 (75–180)	77 (54–143)	**0.001**
LVEDV (mL)	97 (78–153)	80 (58–140)	**0.004**
LV stroke volume (mL)	59 (50–94)	48 (33–87)	**0.001**
LA diameter (mm)	38 (28–45)	33 (25–43)	**0.014**
LA volume (mL)	45 (34–70)	31 (21–59)	**<0.001**
RVD1 (mm)	39 (32–47)	35 (27–46)	**0.005**
RA diameter (mm)	38 (27–48)	35 (26–48)	**0.028**
RA area (cm^2^)	16 (10–22)	12 (8–19)	**0.002**

Data are presented as median with range (min-max). Bold styling denotes statistical significance. LVID, left ventricular internal diameter in diastole; LVPWT, left ventricular posterior wall thickness in diastole; LVM, left ventricular mass; LVEDV, left ventricular end-diastolic volume; LA, left atrium; RVD1, right ventricular basal internal diameter in diastole; RA, right atrium.

**Table 4:** Bivariate correlation of cardiac dimensions vs hormones, all participants.

	Cortisol	IGF-1	IGF-2	FSH	GH	LH	Prolactin	TSH
Peak VO_2_	0.01	0.23	0.08	0.06	−0.12	0.03	0.20	0.02
LVID	0.01	**0.42** ^*∗*^	−0.001	0.24	−0.21	0.16	0.05	−0.09
LVPWT	0.05	0.27	0.03	0.05	−0.13	0.01	0.23	0.10
LVM	0.02	**0.34** ^*∗*^	0.02	0.12	−0.17	0.11	0.12	−0.04
LA diameter	0.001	**0.29** ^*∗*^	−0.07	**0.29** ^*∗*^	−0.13	0.11	−0.05	0.003
LA volume	0.01	**0.31** ^*∗*^	0.004	0.18	−0.26	−0.04	0.14	0.05
RVD1	0.07	0.22	−0.15	0.00	−0.22	0.02	−0.06	−0.11
RA diameter	0.03	**0.38** ^*∗*^	−0.07	−0.09	−0.14	−0.14	−0.04	−0.14
RA area	−0.01	**0.37** ^*∗*^	−0.08	−0.03	−0.12	−0.12	−0.02	−0.11

Data are expressed with Pearson's correlation coefficient. The asterisk ^*∗*^ and bold styling denotes statistical significance <0.05. VO_2_, oxygen uptake. For hormone and cardiac dimensions abbreviation, see previous tables.

**Table 5:** Linear regression between cardiac variables and IGF-1 for active group and controls, respectively.

Cardiac variables	Beta (*P* value)		Beta (*P* value) controlled for BSA	
	Active group	Controls	Active group	Controls
Peak VO2	0.19 (0.238)	0.11 (0.565)	0.18 (0.260)	0.10 (0.651)
LVID	0.10 (0.185)	**0.21 (0.014)**	0.09 (0.116)	0.06 (0.313)
LVPWT	0.01 (0.769)	**0.05 (0.018)**	0.01 (0.861)	0.02 (0.276)
LVM	0.40 (0.617)	**1.52 (0.005)**	0.24 (0.638)	0.63 (0.103)
LA diameter	0.14 (0.168)	0.11 (0.294)	0.12 (0.156)	−0.02 (0.810)
LA volume	0.32 (0.207)	0.27 (0.205)	0.27 (0.094)	−0.04 (0.821)
RVD1	−0.02 (0.879)	0.17 (0.089)	−0.03 (0.706)	0.04 (0.660)
RA diameter	0.09 (0.493)	**0.33 (0.006)**	0.07 (0.519)	0.20 (0.088)
RA area	0.07 (0.377)	**0.16 (0.010)**	0.06 (0.321)	0.06 (0.220)

Beta, unstandardized coefficient. *P* value in parentheses. Bold styling denotes statistical significance. For abbreviations, see previous tables.

## Data Availability

The data used to support the findings of this study are available from the corresponding author upon request.
